# Programmed Cell Death Induced by (−)-8,9-Dehydroneopeltolide in Human Promyelocytic Leukemia HL-60 Cells under Energy Stress Conditions

**DOI:** 10.3390/md12115576

**Published:** 2014-11-20

**Authors:** Haruhiko Fuwa, Mizuho Sato, Makoto Sasaki

**Affiliations:** Graduate School of Life Sciences, Tohoku University, 2-1-1 Katahira, Aoba-ku, Sendai 980-8577, Japan; E-Mails: b3bm1016@s.tohoku.ac.jp (M.S.); masasaki@m.tohoku.ac.jp (M.S.)

**Keywords:** macrolide, apoptosis, programmed cell death, mitochondria, cytotoxicity, energy stress

## Abstract

(+)-Neopeltolide is a marine macrolide natural product that exhibits potent antiproliferative activity against several human cancer cell lines. Previous study has established that this natural product primarily targets the complex III of the mitochondrial electron transport chain. However, the biochemical mode-of-actions of neopeltolide have not been investigated in detail. Here we report that (−)-8,9-dehydroneopeltolide (8,9-DNP), a more accessible synthetic analogue, shows potent cytotoxicity against human promyelocytic leukemia HL-60 cells preferentially under energy stress conditions. Nuclear morphology analysis, as well as DNA ladder assay, indicated that 8,9-DNP induced significant nuclear condensation/fragmentation and DNA fragmentation, and these events could be suppressed by preincubating the cells with a pan-caspase inhibitor, *N*-benzyloxycarbonyl-Val-Ala-Asp(OMe)-fluoromethylketone (zVAD). Immunoblot analysis demonstrated the release of cytochrome *c* from the mitochondria and the cleavage of full-length caspase-3 and poly(ADP-ribose) polymerase (PARP). These results indicated that 8,9-DNP induced caspase-dependent apoptotic programmed cell death under energy stress conditions. It was also found that 8,9-DNP induced non-apoptotic cell death in the presence/absence of zVAD under energy stress conditions. Immunoblot analysis showed the intracytosolic release of apoptosis-inducing factor (AIF), although it did not further translocate to the nucleus. It appears most likely that, in the presence of zVAD, 8,9-DNP triggered necrotic cell death as a result of severe intracellular ATP depletion.

## 1. Introduction

**Figure 1:**
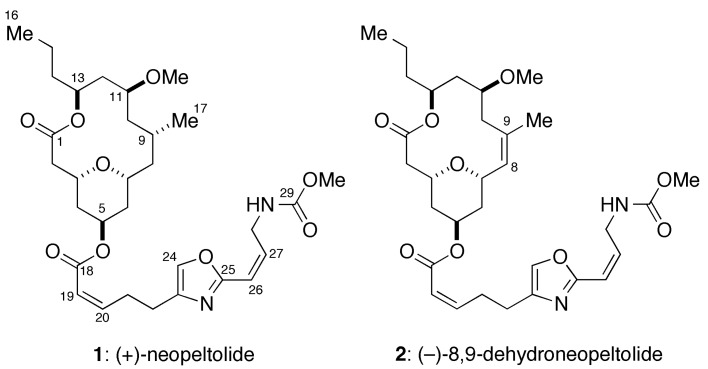
Structures of (+)-neopeltolide (**1**) and (−)-8,9-dehydroneopeltolide (8,9-DNP, **2**).

(+)-Neopeltolide (**1**, Figure 1), a 14-membered macrolide natural product, was isolated from a deep-water sponge that belongs to the family Neopeltidae, collected off the coast of Jamaica [1]. The gross structure, including relative configuration of this natural product was assigned on the basis of extensive 2D NMR analyses. Later, Panek [2] and Scheidt [3,4] independently reassigned the relative configuration and simultaneously established the absolute configuration of **1** through total synthesis. Wright and co-workers have reported potent antiproliferative activity of **1** against several human cancer cell lines, including the A549 human lung adenocarcinoma and the NCI/ADR-RES human ovarian sarcoma cell lines [1]. In addition, Wright *et al*. have described that **1** exhibits antifungal activity against pathogenic yeast *Candida albicans* [1]. Kozmin and co-workers have established that **1** binds to the complex III of the mitochondrial electron transport chain (mETC) as primary molecular target [5]. However, the biochemical mode-of-action and cellular effect of **1** and its synthetic analogues remain largely underexplored.

We have previously reported the total synthesis of **1** and its synthetic analogues to elucidate the structure-activity relationships in detail [6,7,8,9,10]. During our synthetic campaign, we identified the pharmacophoric elements of **1** and discovered several highly potent and synthetically accessible analogues, as exemplified by (−)-8,9-dehydroneopeltolide (8,9-DNP, **2**) [9,11]. Here we report that 8,9-DNP induced caspase-dependent apoptotic cell death in HL-60 human promyelocytic leukemia cells under energy stress conditions. We also describe that 8,9-DNP triggered necrotic death in HL-60 cells when caspase-dependent apoptotic pathway was impaired.

## 2. Results

### 2.1. 8,9-DNP Exhibits Cytotoxicity against HL-60 Cells under Energy Stress Conditions

**Figure 2:**
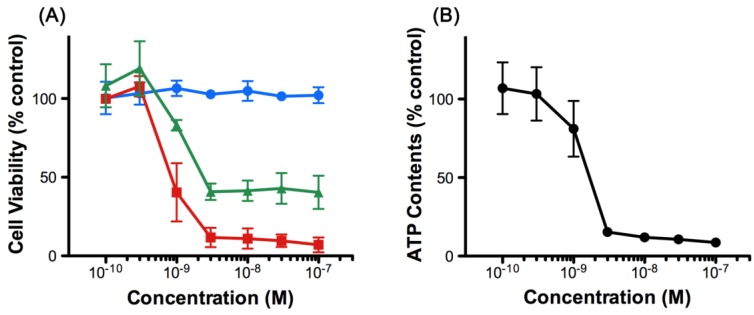
(**A**) Viability of HL-60 cells treated with 8,9-DNP for 24 h. Blue circles: normal medium; green triangles: normal medium + 10 mM 2-DG; red squares: glucose-deprived medium. Data are expressed as mean ± SD of three independent experiments; (**B**) Intracellular ATP concentration of HL-60 cells treated with 8,9-DNP in glucose-deprived medium for 6 h. Data are expressed as mean ± SD of three independent experiments.

The effect of 8,9-DNP in human leukemic cells has not been reported so far. Initially, we examined the viability of HL-60 cells upon treatment with various concentrations of 8,9-DNP by WST-8 assay [12] (Figure 2A). We found that 8,9-DNP did not show appreciable antiproliferative activity against cells cultured in normal medium for 24 h. In contrast, the cell viability decreased when cells were treated with 8,9-DNP under energy stress conditions. Specifically, the cell viability fell below 50% by combined treatment of cells with 8,9-DNP and a glycolysis inhibitor, 2-deoxy-d-glucose (2-DG, 10 mM), in normal medium. Furthermore, marked dose-dependent decrease in cell viability was observed when cells were treated with 8,9-DNP in glucose-deprived medium. These results showed that the cells became sensitized to 8,9-DNP upon inhibition of glycolysis. We also determined that intracellular ATP concentration of HL-60 cells decreased significantly upon exposure to 8,9-DNP in glucose-deprived medium for 6 h (Figure 2B). Hereafter, we investigated the effect of 8,9-DNP on HL-60 cells in glucose-deprived medium as an *in vitro* model of energy stress conditions.

**Figure 3:**
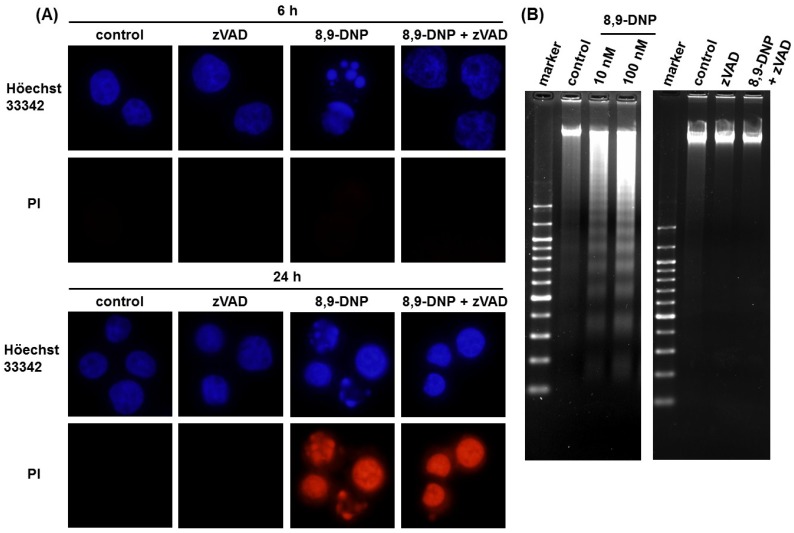
(**A**) Nuclear morphology analysis by Höechst 33342/PI double staining. Each experiment was performed independently two times. Nuclear fragmentation was observed for the cells treated with 8,9-DNP (35% at 6 h and 46% at 24 h); (**B**) DNA electrophoresis on 2% agarose gel, developed with ethidium bromide. Each experiment was performed independently three times.

Next, we performed nuclear morphology analysis using Höechst 33342/propidium iodide (PI) double staining technique (Figure 3A). After treatment of HL-60 cells with 8,9-DNP (100 nM) in glucose-deprived medium for 6 h, a significant portion (35%) of the cells showed nuclear fragmentation without PI staining. A similar result was obtained when cells were treated with 8,9-DNP (100 nM) in the presence of 2-deoxy-D-glucose (10 mM) in normal medium for 6 h (data not shown). In addition, the nuclear fragmentation was suppressed by pretreatment of cells with a pan-caspase inhibitor, *N*-benzyloxycarbonyl-Val-Ala-Asp(OMe)-fluoromethylketone (zVAD) (25 µM) [13,14]. These results were indicative of apoptotic programmed cell death. Meanwhile, almost all the cells treated with 8,9-DNP (100 nM) became PI positive after 24 h in glucose-deprived medium even in the presence of zVAD, whereas control cells did not show appreciable morphological changes and remained PI negative.

Significant DNA laddering was observed in the cells treated with 8,9-DNP under energy stress conditions, which was prevented by co-incubation with zVAD (25 µM) (Figure 3B). These results suggested that internucleosomal cleavage of DNA, one of the characteristics of apoptosis, occurred by the action of 8,9-DNP.

### 2.2. Apoptotic Cell Death Induced by 8,9-DNP Depends on the Intrinsic Pathway

Because it has been shown that neopeltolide inhibits the complex III of isolated mitochondria [5], we envisioned that the apoptotic cell death induced by 8,9-DNP in HL-60 cells might be attributed to the intrinsic mitochondrial pathway [15,16,17], which is known to involve the release of cytochrome *c* (Cyt *c*) from the intermembrane space of the mitochondria to the cytosol [18,19], the activation of caspase-3 by proteolytic processing [20], and the cleavage of poly(ADP-ribose) polymerase (PARP) as characteristic biochemical events [21].

**Figure 4:**
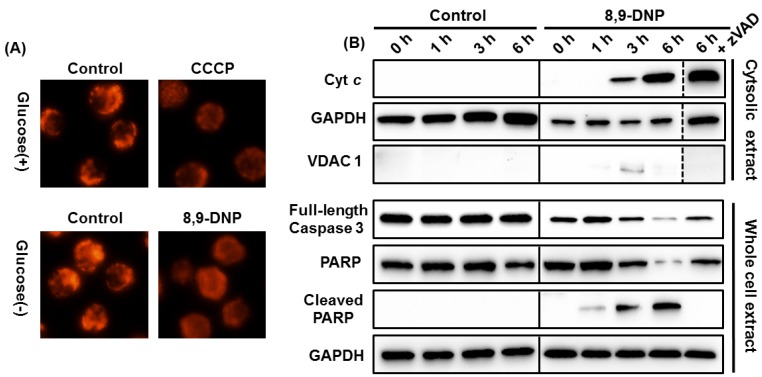
(**A**) Evaluation of ΔΨ_m_ by rhodamine 123 staining. Each experiment was performed independently two times; (**B**) Immunoblot analysis of cell extracts to detect intracytosolic release of Cyt *c* and cleavage of full-length caspase-3 and PARP. The cytosolic extract was probed with anti-VDAC1 antibody to ensure that it was free of mitochondrial component. Each experiment was performed independently three times.

Initially, we assessed the inhibition of the mETC with 8,9-DNP by using rhodamine 123 as a fluorescent probe sensitive to mitochondrial membrane potential (ΔΨ_m_) [22] (Figure 4A). Carbonyl cyanide *m*-chlorophenyl hydrazone (CCCP), an uncoupler of mETC, was used as a positive control. While the untreated cells showed bright punctate signals, the cells treated with 8,9-DNP (100 nM) only showed a dispersed pattern of weak fluorescence, indicating depolarization of ΔΨ_m_. This result confirmed the inhibition of the mETC of HL-60 cells with 8,9-DNP.

Next, we performed immunoblot analysis to gain insight into the mode-of-action of 8,9-DNP. As summarized in Figure 4B, the cytosolic extract of the HL-60 cells treated with 8,9-DNP (100 nM) in glucose-deprived medium for different periods of time (1, 3, and 6 h) showed the presence of cytosolic Cyt *c* after 3 h. Furthermore, in the whole cell extract, the decrease of full-length caspase-3 (procaspase-3) and the cleavage of PARP were observed after 3 h. The PARP cleavage was abolished by co-incubation with zVAD (25 µM) prior to 8,9-DNP treatment. Taken together, these results strongly suggested that the release of Cyt *c* from the mitochondria, triggered by 8,9-DNP, induced apoptotic cell death via the intrinsic pathway involving caspase activation and PARP cleavage.

### 2.3. 8,9-DNP also Induces Intracytosolic Release of AIF but It Does Not Translocate to the Nucleus

The results of the nuclear morphology analysis, DNA laddering experiments, and immunoblot analysis clearly indicated that zVAD successfully inhibited caspases in the cells treated with 8,9-DNP under energy stress conditions and rescued the cells from apoptotic death. However, Höechst 33342/PI double staining analysis showed that zVAD could not prevent cell death after 24 h treatment with 8,9-DNP (Figure 3A). Neither nuclear fragmentation nor condensation were apparent for the cells treated with 8,9-DNP in the presence of zVAD in glucose-deprived medium for 24 h. Nonetheless, all the cells were clearly stained with PI.

**Figure 5:**
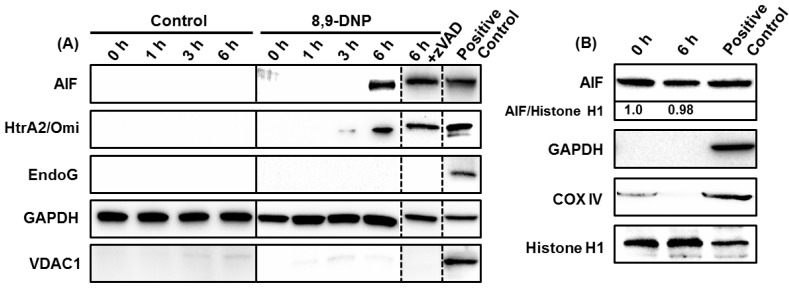
(**A**) Immunoblot analysis of cytosolic extract showed intracytosolic release of AIF and HtrA2/Omi, but not EndoG. The cytosolic extract was also probed with anti-VDAC1 antibody to ensure that it was free of mitochondrial proteins. The whole cell extract of untreated cells was used as positive control; (**B**) Immunoblot analysis of nuclear extract showed that AIF did not translocate to the nucleus. The nuclear extract was also probed with anti-GAPDH and anti-COX IV antibodies to ensure that it was free of cytosolic and mitochondrial proteins. The whole cell extract of untreated cells was used as positive control. Each experiment was performed independently three times.

Accordingly, we performed further immunoblot analysis of the cytosolic extract of the cells treated with 8,9-DNP in glucose-deprived medium for different periods of time (1, 3, and 6 h). Apoptosis-inducing factor (AIF) [23,24] and endonuclease G (Endo G) [25,26] are known to induce caspase-independent programmed cell death upon translocation to the nucleus. High-temperature requirement protein A2 (HtrA2)/Omi [27] is a serine protease that contributes to apoptosis by antagonizing inhibitor of apoptosis (IAP) proteins. The intracytosolic release of AIF and HtrA2/Omi, but not Endo G, was observed after 6 h, regardless of the presence of zVAD (Figure 5A). These results indicated that the intracytosolic release of AIF and HtrA2/Omi was specifically induced by the action of 8,9-DNP and that zVAD did not block the release of AIF and HtrA2/Omi to the cytosol but merely inhibited downstream caspase-dependent events. At this stage, it was envisaged that AIF might be a primary executor of the caspase-independent cell death in 8,9-DNP-treated cells. However, translocation of AIF to the nucleus was not detected by immunoblot analysis of the nuclear extract of the cells exposed to 8,9-DNP in glucose-deprived medium for 6 h (Figure 5B). This result indicated that AIF is not responsible for the caspase-independent cell death induced by 8,9-DNP.

## 3. Discussion

The mitochondrial electron transport chain (mETC), consists of four transmembrane complexes, I, II, III, and IV, generates electrochemical proton gradient across the inner membrane of the mitochondria to facilitate ATP production by F_1_F_o_ ATP synthase (complex V). The mitochondrial complex III is known to transfer electrons from ubiquinol to Cyt *c* and pump protons from the inner matrix to the intermembrane space using the Q-cycle. Naturally occurring antibiotics, such as antimycin A, stigmatellin, and myxothiazol, have contributed to the characterization of the structure and functions of the mitochondrial complex III at the molecular level [28,29]. It has been shown that antimycin A binds to the Q_o_-site, whereas stigmatellin and myxothiazol bind to the Q_i_-site [28,29]. (+)-Neopeltolide is a new potent inhibitor of the complex III of the mETC, recently identified from a deep-water sponge of the family Neopeltidae [1,5]. However, the precise location of its binding site has not been established.

The mitochondria play a key role in programmed cell death [16,17]. In response to apoptotic stimuli, the mitochondria release Cyt *c* from the intermembrane space to the cytosol via outer membrane permeabilization. Cyt *c* binds to apoptotic protease activating factor-1 (Apaf-1) in the cytosol to form apoptosome, which triggers activation of the caspase cascade [18,19]. Several mitochondrial proteins, such as AIF, EndoG, and HtrA2/Omi, are also known to induce programmed cell death [20,21,22,23,24,25,26,27].

In this study, we investigated the effect of (−)-8,9-DNP [9,11], a potent synthetic analogue of (+)-neopeltolide, on HL-60 human promyelocytic leukemia cells. We found that 8,9-DNP decreased cell viability preferentially under the conditions in which glycolytic ATP generation was inhibited by 2-deoxy-D-glucose or by withdrawal of D-glucose from culture medium (Figure 2). Clearly, the inhibition of glycolysis made the cells significantly sensitive to 8,9-DNP [30,31]. Under these energy stress conditions, internucleosomal DNA fragmentation occurred significantly in the cells treated with 8,9-DNP, as indicated by the nuclear morphology characterization and DNA laddering experiment (Figure 3). These phenotypes are the hallmarks of caspase-dependent programmed cell death [15,16,17], and they were specifically suppressed by preincubating cells with a pan-caspase inhibitor zVAD [13,14]. Furthermore, immunoblot analysis of the cells treated with 8,9-DNP in glucose-deprived medium showed the leakage of Cyt *c*, an apoptogenic protein, from the intermembrane space of the mitochondria to the cytosol, the activation of caspase-3, and the cleavage of PARP (Figure 4B). Collectively, these results indicated that the cells exposed to 8,9-DNP under energy stress conditions underwent caspase-dependent apoptotic programmed cell death via the mitochondrial intrinsic pathway.

Additional immunoblot analysis showed that the intracytosolic release of AIF and HtrA2/Omi occurred after 6 h treatment of cells with 8,9-DNP in glucose-deprived medium (Figure 5A). Presumably, the release of these mitochondrial proteins could be ascribed to mitochondrial outer membrane permeabilization induced by the loss of ΔΨ_m_ [32]. Moreover, prolonged treatment of HL-60 cells with 8,9-DNP induced cell death even in the presence of zVAD. We initially speculated that AIF might be responsible for the observed cell death, since AIF is known to contribute to programmed cell death when caspases are inhibited or not activated [32]. Ogita *et al*. have recently reported that antimycin A, a potent complex III inhibitor, induces nuclear translocation of AIF in a caspase 3-independent manner to show cytotoxicity against HL-60 cells [33]. However, in the present case, nuclear translocation of AIF was not observed (Figure 5B). Thus, it is most likely that AIF is not responsible for the caspase-independent cell death triggered by 8,9-DNP.

Meanwhile, it is known that ATP is required for executing caspase-dependent apoptotic cell death and that severe intracellular ATP depletion causes necrotic, programmed cell death [34,35]. In addition, inhibitors of the mETC have been shown to be cytotoxic against the PANC-1 human pancreatic carcinoma cell line under nutrient-deprived conditions, where marked decrease of intracellular ATP concentration was observed [36]. Actually, intracellular ATP concentration of HL-60 cells considerably decreased after 6 h treatment with 8,9-DNP in glucose-deprived medium (Figure 2B), implying that the cells might undergo necrotic programmed cell death because of energetic impairment induced by dual inhibition of glycolysis and oxidative phosphorylation (OXPHOS).

Because of metabolic requirements for rapid proliferation, the energy metabolism of cancer cells depends heavily on glycolysis even when sufficient oxygen is available (“aerobic glycolysis”), and hence differs significantly from that of normal cells [37,38,39]. It is known that leukemic cells are highly glycolytic [40], and that inhibition of the glycolytic activity sensitizes them to chemotherapeutic agents [30,31,41,42]. It has been reported that the combination of 2-deoxy-d-glucose and an AMP-activated protein kinase (AMPK) agonist metformin is effective against human solid tumor cells [43,44] and leukemic cells [45] *in vitro* and *in vivo*. Metformin compromises mitochondrial ATP synthesis by inhibiting complex I of the mETC and depolarizing ΔΨ_m_ [43,44,45]. Thus, dual inhibition of glycolysis and OXPHOS is an effective means to target the bioenergetics of cancer cells. It would be of interest to investigate whether 8,9-DNP shows cytotoxicity against solid tumor cells under energy stress conditions, because cancer cells in tumor microenvironment are often exposed to low-nutrient, hypoxic conditions due to their rapid proliferation and insufficient angiogenesis within solid tumors [46]. Work toward this end is underway.

## 4. Experimental Section

### 4.1. Materials

The HL-60 human promyelocytic leukemia cell line was provided from RIKEN BRC through the National Bio-Resource Project of the MEXT, Japan. Primary antibodies were obtained as follows: rabbit anti-caspase-3 monoclonal antibody, rabbit anti-PARP monoclonal antibody, rabbit anti-COX IV monoclonal antibody (Cell Signaling Technology, Danvers, MA, USA); rabbit anti-AIF polyclonal antibody, mouse anti-histone H1 monoclonal antibody, and mouse anti-VDAC1 monoclonal antibody (Santa Cruz Biotechnology, Dallas, TX, USA); mouse anti-Cyt *c* monoclonal antibody (BD Biosciences, San Jose, CA, USA); rabbit anti-endonuclease G polyclonal antibody (EMD Millipore, Temecula, CA, USA); mouse anti-HtrA2/Omi monoclonal antibody (R&D Systems, Minneapolis, MN, USA); HRP-conjugated mouse anti-GAPDH monoclonal antibody (MBL, Nagoya, Japan). Höechst 33342 and propidium iodide were obtained from Life Technologies (Carlsbad, CA, USA). zVAD was purchased from Peptide Institute (Osaka, Japan). (−)-8,9-Dehydroneopeltolide (8,9-DNP) was synthesized as described previously [9] and purified by preparative high-performance liquid chromatography performed on a Shimadzu Prominence HPLC System equipped with a COSMOSIL 5C18AR-II column (250 mm × 20 mm I.D., Nacalai Tesque, Kyoto, Japan). All other chemicals were purchased from Nacalai Tesque (Kyoto, Japan), Wako Pure Chemicals (Osaka, Japan), Dojindo Laboratories (Kumamoto, Japan), or Sigma-Aldrich (St. Louis, MO, USA). All cell culture media used in this study were supplemented with 10% fetal bovine serum, 100 units/mL of penicillin, and 100 µg/mL of streptomycin and filter-sterilized before use.

### 4.2. Cell Viability Assay

Cells were cultured for 2–3 days in RPMI1640 medium maintaining under a 5% CO_2_/air atmosphere in a CO_2_ incubator at 37 °C. Exponentially growing cells were harvested and re-suspended in RPMI1640, RPMI1640 containing 10 mM 2-deoxy-d-glucose, or RPMI1640 without d-glucose at a density of 2.5 × 10^5^ cells/mL. The cell suspension (198 µL) was distributed to wells of a 96-well microtiter plate. The cells were then treated with various concentrations of 8,9-DNP (2 µL) and incubated in a CO_2_ incubator at 37 °C for 24 h. At this point, WST-8 (5 µL) was added to each well. After incubation in a CO_2_ incubator at 37 °C for additional 5 h, the UV absorbance at 405 nm and 650 nm, respectively, was measured by a microplate reader (Synergy^®^HT Multi-Mode Microplate Reader, BioTek Instruments, Winooski, VT, USA). The relative absorbance values, calculated by subtracting UV absorbance at 650 nm from that at 450 nm, were plotted against sample concentrations (the relative absorbance values for 0% and 100% cell viability were obtained with blank and 10% MeOH/H_2_O, respectively). IC_50_ values were calculated with a non-linear regression model of standard slope by using the GraphPad Prism software (GraphPad Software, La Jolla, CA, USA).

### 4.3. Measurement of Intracellular ATP Concentration

Intracelluar ATP concentration of HL-60 cells was determined by using CellTiter-Glo assay kit (Promega, Madison, WI, USA) according to the manufacturer’s protocol.

### 4.4. Nuclear Morphology Analysis

The Höechst 33342/propidium iodide double staining technique was used to observe nuclear morphology and to discriminate living and dead cells. In cases where zVAD was used as an additive, cells were incubated with zVAD (25 µM) at 37 °C for 30 min prior to 8,9-DNP treatment. After treatment of HL-60 cells with 8,9-DNP (100 nM) in RPMI1640 without d-glucose at 37 °C for 6 or 24 h, the cells were harvested, washed once with PBS, and then treated with 10 µg/mL Höechst 33342 and 5 µg/mL propidium iodide in PBS at room temperature for 30 min. The cells were washed once with PBS and fixed with 4% paraformaldehyde in PBS at room temperature for 20 min. The cells were washed once with PBS and then observed under an inverted fluorescence microscope (IX-73, Olympus, Tokyo, Japan) equipped with a 60× objective.

### 4.5. Mitochondrial Membrane Potential Assay

After treatment of exponentially growing HL-60 cells with 8,9-DNP (100 nM) in glucose-deprived medium for 1 h, the cells were washed with PBS and stained with rhodamine 123 (10 µg/mL) in PBS at room temperature for 15 min. The cells were washed with PBS, and observed under an inverted fluorescence microscope (IX-73, Olympus, Tokyo, Japan) equipped with a 40× objective.

### 4.6. DNA Laddering Assay

Exponentially growing HL-60 cells were treated with 8,9-DNP (100 nM) in RPMI1640 without d-glucose at 37 °C for 6 h. DNA was extracted from the cells by using SepaGene kit (Eidia, Tokyo, Japan) according to the manufacturer’s protocol. The extracted DNA was dissolved in 20 µg/mL RNase/TE buffer, incubated at 37 °C for 30 min, and then electrophoresed on a 2% agarose gel in TAE buffer. The gel was stained with 1 µg/mL ethidium bromide, rinsed with distilled water, and then visualized under UV light (ChemiDoc XRS+ Imaging System, Bio-Rad, Hercules, CA, USA).

### 4.7. Preparation of Cytosolic Extract

After treatment of exponentially growing HL-60 cells with 8,9-DNP (100 nM) in RPMI1640 medium without d-glucose at 37 °C for 1, 3, or 6 h, cells were harvested, washed once with PBS, and then incubated on ice for 5 min with a buffer containing 75 mM KCl, 1 mM NaH_2_PO_4_, 8 mM Na_2_HPO_4_, 250 mM sucrose, 200 µg/mL digitonin, and protease inhibitor cocktail (Nacalai Tesque, Kyoto, Japan). After centrifugation (1500× *g*, 4 °C, 5 min; then 16,000× *g*, 4 °C, 5 min), the supernatant was saved as the cytosolic extract and stored at −80 °C before use. Protein concentration was determined by using Protein Assay Rapid kit (Wako Pure Chemical, Osaka, Japan).

### 4.8. Preparation of Whole Cell Extract

After treatment of exponentially growing HL-60 cells with 8,9-DNP (100 nM) in RPMI1640 medium without d-glucose at 37 °C for 1, 3, or 6 h, cells were harvested, washed once with PBS, and then incubated on ice with RIPA buffer (Wako Pure Chemicals, Osaka, Japan) supplemented with protease inhibitor cocktail (Nacalai Tesque, Kyoto, Japan) for 30 min. After centrifugation (10,000× *g*, 4 °C, 10 min), the supernatant was saved as the whole cell extract and stored at −80 °C before use. Protein concentration was determined by using Protein Assay Rapid kit (Wako Pure Chemical, Osaka, Japan).

### 4.9. Preparation of Nuclear Extract

HL-60 cells treated with or without 8,9-DNP (100 nM) were harvested, washed twice with ice-cold PBS, and incubated with ice-cold hypotonic buffer (10 mM HEPES/KOH (pH 7.9), 2 mM MgCl_2_, 0.1 mM EDTA, 10 mM KCl, 1 mM DTT, and 0.5% Triton X-100) supplemented with protease inhibitor cocktail (Nacalai Tesque, Kyoto, Japan) on ice for 10 min. After centrifugation (800× *g*, 4 °C, 8 min), the supernatant was removed and the pellet gently suspended in the ice-cold hypotonic buffer. After centrifugation (800× *g*, 4 °C, 8 min), the supernatant was removed and the pellet gently suspended in SDS buffer (0.1 M Tris∙HCl (pH 6.8), 2% SDS, and 20% glycerol) supplemented with protease inhibitor cocktail (Nacalai Tesque, Kyoto, Japan). The resultant solution was briefly sonicated in an ice-cold bath, diluted with an equal volume of 200 mM DTT plus 0.04% bromophenol blue, and boiled at 95 °C for 5 min. The nuclear extract thus obtained was stored at −80 °C before use.

### 4.10. Immunoblot Analysis

The cellular extract prepared above was diluted with 2× Sample Buffer Solution (Nacalai Tesque, Kyoto, Japan) containing 100 mM DTT and boiled at 95 °C for 3 min. After cooling on ice, the resultant mixture was resolved on a 12% SDS-polyacrylamide gel and then transferred onto a PVDF membrane. The membrane was blocked with Blocking One (Nacalai Tesque, Kyoto, Japan) at room temperature for 30 min and probed with an appropriate primary antibody at room temperature for 1–4 h or at 4 °C overnight. The membrane was then probed with an appropriate HRP-conjugated secondary antibody at room temperature for 1 h and then developed with Chemi-Lumi One (Nacalai Tesque, Kyoto, Japan). Chemiluminescence was detected on a ChemiDoc XRS+ Imaging System (Bio-Rad, Hercules, CA, USA).

## 5. Conclusions

This study has demonstrated that 8,9-DNP, a potent synthetic analogue of (+)-neopeltolide, induces apoptotic cell death in HL-60 human promyelocytic leukemia cells under energy stress conditions. The mechanism of the apoptotic cell death involves the release of Cyt *c* from the mitochondria to the cytosol, activation of caspase-3, cleavage of PARP, and DNA fragmentation. The apoptotic cell death induced by 8,9-DNP could be suppressed by co-incubation of cells with a pan-caspase inhibitor zVAD. It was also found that 8,9-DNP caused non-apoptotic cell death in the presence of zVAD. Immunoblot analysis of the cytosolic extract of the cells exposed to 8,9-DNP showed the release of AIF and HtrA2/Omi from the intermembrane space of the mitochondria to the cytosol, although the nuclear level of AIF did not increase under these conditions. It seems most likely that significant decrease of intracellular ATP concentration caused by 8,9-DNP resulted in necrotic cell death where caspases were inhibited or not activated. Further studies on the biological activity and mode-of-action of 8,9-DNP are currently underway and will be reported shortly.
